# Multiplex Microscopy Assay for Assessment of Therapeutic and Serum Antibodies against Emerging Pathogens

**DOI:** 10.3390/v16091473

**Published:** 2024-09-17

**Authors:** Nuno Sartingen, Vanessa Stürmer, Matthias Kaltenböck, Thorsten G. Müller, Paul Schnitzler, Anna Kreshuk, Hans-Georg Kräusslich, Uta Merle, Frauke Mücksch, Barbara Müller, Constantin Pape, Vibor Laketa

**Affiliations:** 1 Department of Infectious Diseases, Virology, Medical Faculty Heidelberg, Heidelberg University, 69120 Heidelberg, Germany; nuno.sartingen@stud.uni-heidelberg.de (N.S.); vanessa.stuermer@embl.de (V.S.); matthiaskaltenboeck@gmail.com (M.K.); muellert@ie-freiburg.mpg.de (T.G.M.); paul.schnitzler@med.uni-heidelberg.de (P.S.); hans-georg.kraeusslich@med.uni-heidelberg.de (H.-G.K.); frauke.muecksch@med.uni-heidelberg.de (F.M.); barbara.mueller@med.uni-heidelberg.de (B.M.); 2 European Molecular Biology Laboratory, 69117 Heidelberg, Germany; anna.kreshuk@embl.de; 3 German Center for Infection Research (DZIF), Partner Site Heidelberg, 69120 Heidelberg, Germany; 4 Department of Internal Medicine IV, University Hospital Heidelberg, 69120 Heidelberg, Germany; uta.merle@med.uni-heidelberg.de; 5 Institute of Computer Science, Göttingen University, 37073 Göttingen, Germany; constantin.pape@informatik.uni-goettingen.de; 6 Cluster of Excellence ‘Multiscale Bioimaging: from Molecular Machines to Networks of Excitable Cells’ (MBExC), Göttingen University, 37073 Göttingen, Germany

**Keywords:** multiplex microscopy, machine learning, serology, emerging pathogens, monoclonal antibodies, SARS-CoV-2

## Abstract

The emergence of novel pathogens, exemplified recently by the severe acute respiratory syndrome coronavirus 2 (SARS-CoV-2), highlights the need for rapidly deployable and adaptable diagnostic assays to assess their impact on human health and guide public health responses in future pandemics. In this study, we developed an automated multiplex microscopy assay coupled with machine learning-based analysis for antibody detection. To achieve multiplexing and simultaneous detection of multiple viral antigens, we devised a barcoding strategy utilizing a panel of HeLa-based cell lines. Each cell line expressed a distinct viral antigen, along with a fluorescent protein exhibiting a unique subcellular localization pattern for cell classification. Our robust, cell segmentation and classification algorithm, combined with automated image acquisition, ensured compatibility with a high-throughput approach. As a proof of concept, we successfully applied this approach for quantitation of immunoreactivity against different variants of SARS-CoV-2 spike and nucleocapsid proteins in sera of patients or vaccinees, as well as for the study of selective reactivity of monoclonal antibodies. Importantly, our system can be rapidly adapted to accommodate other SARS-CoV-2 variants as well as any antigen of a newly emerging pathogen, thereby representing an important resource in the context of pandemic preparedness.

## 1. Introduction

The emergence of the novel pathogenic severe acute respiratory syndrome coronavirus 2 (SARS-CoV-2) in 2019 [1,2] and its rapid pandemic spread had profound repercussions on societies worldwide [3,4,5]. To mitigate the viral transmission and reduce infection rates, governments implemented extensive lockdown measurements and social restrictions. In an effort to restrain the pandemic, the swift development of SARS-CoV-2 vaccines led to a massive vaccination effort with more than 5 billion people vaccinated worldwide as of July 15, 2024 [6,7,8]. However, the continuous evolution of new viral variants led to immune evasion, affecting viral infectivity and pathogenicity [9], and compromised the effectiveness of the first vaccines based on the wild-type Wuhan-Hu-1 sequence of the viral spike protein [10,11]. The spike protein also had a central role in the development of anti-viral treatments based on therapeutic monoclonal antibodies (mAbs) [12,13]. However, with the emergence of novel variants, the initially developed therapeutic mAbs lost their anti-viral efficacy due to mutations in the spike protein [14,15,16]. These lessons learned during the COVID-19 pandemic need to be leveraged to prepare our response to any future pandemics characterized by quickly evolving pathogen variants. In the context of pandemic preparedness, it will be essential to rapidly (i) evaluate the status of the immunity of the population and assure the effectiveness of vaccines and (ii) support the development of new mAb-based anti-viral treatments.

To achieve these objectives, it is essential to develop comprehensive serological assays that can assess antibody binding to different viral proteins and are rapidly adaptable to include emerging variants of concern. In the broader context of public health management and infectious disease control, serological assays are an essential complement to PCR testing. Antibody detection and quantification played a fundamental role in decision-making and policy guidance aimed at restraining the spread of SARS-CoV-2 [17,18] and will be equally important in constraining any pandemic in the future. Currently, commercially available enzyme-linked immunosorbent assays or (electro) chemiluminescent immunoassays (ELISA, (E)CLIA) represent the gold standard for antibody detection. However, adapting them rapidly to new viral variants in an evolving pandemic situation presents challenges, primarily due to the need for protein expression, purification and optimization of biochemical properties. This challenge is especially pronounced for highly glycosylated proteins expressed in cellular and viral membranes [19,20,21,22]. Moreover, standard ELISA assays cannot assess binding to multiple viral proteins simultaneously, effectively limiting their throughput and information content. Multiplex serology for simultaneous detection of antibodies against different SARS-CoV-2 proteins based on protein microarrays [23] or fluorescent beads [24] has been applied during the COVID-19 pandemic. However, these approaches also rely on the availability of purified proteins. Additionally, the recent SARS-CoV-2 pandemic highlighted the importance of alternative serological assays in situations where commercial ELISA supplies are scarce or technically infeasible.

In response to the outlined challenges, we describe the development of an automated multiplex microscopy and machine learning-based serology assay for simultaneous differential antibody detection, focusing on SARS-CoV-2 as a proof of concept. For this, we build upon our previous work, where we established a serology assay for SARS-CoV-2 antibody detection in human samples [25] and employed it as a complementary assay in an early large-scale seroprevalence study [26]. The multiplexed approach presented here provides differentiated data about antibody reactivity against various viral antigens at once and can be conducted within a biosafety level 1 laboratory. By applying this method to screen a cohort of sera from convalescent individuals and vaccinees, we successfully demonstrated its efficacy in determining immunoreactivity against the SARS-CoV-2 nucleocapsid protein and different variants of SARS-CoV-2 spike proteins. Additionally, it enabled the evaluation of selective reactivity of (therapeutic) monoclonal antibodies (mAbs). Notably, the system described here can be swiftly adapted to assess immunoreactivity to new SARS-CoV-2 variants or other antigens from emerging pathogens, making it a valuable tool for pandemic preparedness. It represents a valuable tool in our portfolio of serological assays with the potential to complement and expand diagnostic procedures as well as facilitate the screening and testing of therapeutic antibodies.

## 2. Materials and Methods

### 2.1. Human Material

Negative control serum samples (*n* = 233) were collected for various serological testing in the routine laboratory of the Center of Infectious Diseases, University Hospital Heidelberg between 2015 and 2019, before the start of the SARS-CoV-2 outbreak. Samples used corresponded to pseudonymized material from the Center of Infectious Diseases, Heidelberg. SARS-CoV-2 positive sera were collected from 41 RT-PCR-confirmed symptomatic COVID-19 patients treated at the University Hospital Heidelberg after giving their written informed consent (ethics votum no S-148/2020, University Hospital Heidelberg). Days post-infection were defined based on the anamnesis carried out upon admission. Serum samples were stored at −20 °C until use.

### 2.2. Plasmids

Plasmids were cloned using standard molecular biology techniques and verified by commercial Sanger sequencing (Eurofins Genomics, Ebersberg, Germany). pWPI lentiviral plasmids [27] served as a vector backbone for the insertion of genes of interest. Genes of interest were integrated into the pWPI sequence using classical cloning and Gibson assembly. The assembly was performed using the NEB HiFi Mastermix (New England Biolabs, Ipswich, MA, USA) with 30 base–pair overlap regions. PCR was performed using Q5 High-Fidelity DNA Polymerase (New England Biolabs), according to the manufacturer’s instructions with primers purchased from Eurofins Genomics. E. coli DH5α and Stbl2 [28] (Thermo Fisher Scientific, Waltham, MA, USA) were used for amplification of standard plasmids or LTR-containing plasmids, respectively. The list of plasmids used can be found in Table S1. Schematic representation of the generated plasmids is shown in Figure S1.

### 2.3. Cell Culture

HeLa cells and Hek293T cells were authenticated using STR profiling (Eurofins Genomics) and monitored for mycosplasma contamination using the MycoAlert mycoplasma detection kit (Lonza, Basel, Switzerland). Cells were cultured at 37 °C and 5% CO_2_ in Dulbecco’s Modified Eagle’s Medium (DMEM; Thermo Fisher Scientific) containing 4.5 g/L D-glucose and L-glutamine supplemented with 10% fetal calf serum (FCS; Sigma-Aldrich, St. Louis, MO, USA), 100 U/mL penicillin and 100 µg/mL streptomycin (PAN Biotech, Aidenbach, Germany).

### 2.4. Generation of Cell Lines

A total of 4 × 10^5^ Hek293T cells were seeded per well into 6 well plates and incubated overnight. Lentiviral vector particles were produced by co-transfection of packaging plasmid psPAX2 (Addgene #12260), the respective lentiviral transfer vector pWPI, the envelope expression plasmid pCMV-VSV-G (Addgene #8454) and pAdvantage (Promega, Madison, WI, USA) in a ratio of 1.5:1.0:0.5:0.2 µg into HEK293T cells using polyethylenimine (PEI; 3 µL of 1 mg/mL PEI per µg DNA). At 48 h post-transfection, the supernatant was harvested and filtered through 0.45 µm mixed cellulose ester (MCE) filters, and 500 µL of lentiviral vector particle containing supernatant was added to HeLa cells (5 × 10^4^ cells/well seeded the day before in 12 well plates). After 48 h, the transduced cells were selected using 5 mg/mL blasticidin or 1 mg/mL puromycin and subsequently expanded to a 25 mL or 75 mL cell culture flask depending on the number of cells. After three or five days (for blasticidin and puromycin, respectively) under antibiotic selection, cells were transduced with the respective second construct. Cells were first transduced with lentiviral vector particles encoding the subcellular fluorescent localization marker, followed by transduction with corresponding viral proteins. Cells were cultured under ongoing selection pressure with both antibiotics. Cell lines were cultured separately and mixed in equal numbers at the start of the experiment. The full list of cell lines used and created in this work can be found in Table S2.

### 2.5. Fluorescence-Activated Cell Sorting

After successful double transduction, fluorescence-activated cell sorting (FACS) was employed for additional double sorting of each generated cell line. Cells were suspended in phosphate-buffered saline (PBS). After addition of undiluted accutase (StemPro™ Accutase^®^ Cell Dissociation Reagent, Thermo Fisher Scientific) as per the manufacturer’s instructions, cells were incubated for 15 min at 37 °C. Subsequently, cells were resuspended in PBS and harvested by centrifugation (5 min, 1500 rpm, Heraeus Megafuge 40R (Heraeus, Hanau, Germany)). Sorting of barcoded cell lines was performed based on the expression of the mScarlet-marker. For sorting of spike-expressing cells, additional staining targeting surface-expressed spike proteins was performed. Cells were resuspended in 3 ml PBS and 2% fetal calf serum as blocking buffer and incubated for 15 min. After incubation, a total of 4 × 10^6^ cells per sample were resuspended in a volume of 2 ml PBS and primary anti-IgG1-Wuhan-Hu-1-spike antibody (Biozol, Eching, Germany, Cat# GTX632604) was applied in a dilution of 1:1000. Samples were incubated on ice for 30 min in the dark. Samples were washed with PBS and incubated with polyclonal Alexa Fluor 488 secondary antibody in a dilution of 1:2000 on ice for 20 min in the dark. After washing the samples with PBS, double-positive cells for the mScarlet (568 nm) and viral protein signal (488 nm) were sorted. The nucleocapsid-3xNLS-mSc cell line was only sorted for the mScarlet expression as nucleocapsid is exclusively expressed in the cytosol.

### 2.6. Monoclonal Antibodies

REGN10933 (Casirivimab) and REGN10987 (Imdevimab) were procured in an infusion solution, containing both antibodies at a concentration of 120 mg/mL (Roche, Basel, Switzerland; Swissmedic marketing authorization number 68329). The antibodies were diluted according to the manufacturer’s instructions before use in subsequent experimental procedures. Monoclonal anti-RBD antibodies analyzed in paragraph 3.3 were described previously [29]. Briefly, RBD-specific B cells were isolated from study participants who had been vaccinated with three doses of an mRNA vaccine and reported SARS-CoV-2 Omicron BA.1 breakthrough infection (variant deduced based on prevalent variant circulating in New York City at the time of infection). Antibody sequences were amplified from reverse-transcribed cellular RNA, sequenced, cloned and expressed as recombinant human IgG. The full list of mAbs can be found in Table S3.

### 2.7. Immunofluorescent Staining

Staining was performed in a black-wall glass bottom 96-well plate (Cellvis, Mountain View, CA, USA 96-well glass bottom plate with high-performance #1.5 cover glass). The various HeLa cell lines were seeded at a density of 1.5 × 10^4^ cells per well 16–18 h before staining (5 cell lines = 3 × 10^3^ per cell line/well). After washing with PBS, cells were fixed with 4% paraformaldehyde (PFA) for 20 min followed by 3× washing with PBS. Cells were then permeabilized with 0.5% Triton X100 (Sigma-Aldrich) for 5 min and washed 3 times with PBS containing 0.02% Tween 20 (Sigma-Aldrich) (PBS+). Blocking was carried out with 2% powdered milk (Roth) in PBS+ for 20 min followed by 3 washes with PBS+. Subsequently, cells were incubated with primary antibodies (mouse anti-spike antibody Biozol GTX632604 1:2000 in PBS+, mouse anti-nucleocapsid Sino Biological, Beijing, China, 40143-MM05 1:4000 in PBS+, mAbs with different indicated dilutions) or patient sera (1:200 in PBS+) for 30 min in the dark. Following three washing steps, samples were incubated with corresponding secondary antibodies for 20 min at room temperature in the dark (goat anti-human IgG-AlexaFluor 488 (Invitrogen, Thermo Fisher Scientific) 1:2000, goat anti-mouse IgG 647 1:500). After two washing steps with PBS+, cells were stained with Hoechst 33342 (Thermo Fisher Scientific) at a concentration of 0.2 µg/mL in PBS for 5 min before undergoing three washes with PBS. Samples were stored at 4 °C until imaging. All washing steps in a 96-well format were performed using the HydroFlex microplate washer (Tecan, Männedorf, Switzerland). Living cells were incubated with mAbs (200 ng/mL and 2 ug/mL) for 15 min directly in growth medium at room temperature, followed by two washing steps with PBS and fixation with 4% PFA for 20 min. Further staining was performed as described above.

### 2.8. Microscopy

Images were acquired on the Zeiss CellDiscoverer 7 automated microscope (Zeiss, Oberkochen, Germany) using a Plan-Apochromat 20×/0.95 Air objective with 1× tube lens magnification and a Zeiss Axiocam 712 sCMOS camera for detection. Multichannel images were acquired using 385 nm (Nucleus channel) and 625 nm (Antigen expression channel) light-emitting diodes for excitation and a quad-bandpass filter QBP 425/30 + 514/30 + 592/25 + 709/100 for emission. “IgG channel” and “Barcode channel” were acquired using a 470 nm LED together with 515/25 bandpass emission filter and 567 nm LED with 595/30 bandpass emission filter, respectively. A total of 9–12 images were acquired in each well, spaced in a regular 3 × 3 or 3 × 4 pattern centered around the middle of the well or randomly distributed. A definite focus system was used for axial plane stabilization during imaging.

### 2.9. Enzyme-Linked Immuno-Sorbent Assay (ELISA)

ELISA measurements to determine reactivity against the S1 domain of the viral spike protein were conducted using the Euroimmun Anti-SARS-CoV-2-ELISA (IgG) test kits (Euroimmun, Lübeck, Germany; and EI 2606-9601 451 G). The assays were run on a Euroimmun Analyzer I instrument following the manufacturer’s instructions. Optical densities measured for the samples were normalized using the value obtained for a calibrator sample provided in the test kit. The interpretation of the semi-quantitative ratiometric values were obtained following the manufacturer’s protocol: values < 0.8 RU/mL were classified as negative, 0.8–1.1 RU/mL as borderline, and values of 1.1 RU/mL or higher as positive.

### 2.10. Image Analysis

Cell segmentation was adapted from Pape et al. [25] and was based on a seeded watershed using segmented nuclei as seeds and boundary predictions as the heightmap. For nucleus segmentation, the pretrained “Fluo” model of StarDist [30] was used. It was qualitatively checked and shows high prediction quality on our data. For boundary prediction, we used a U-Net [31] applied to the IgG channel, starting from the model trained in Pape et al. [25]. However, due to the different appearance of this channel, the model exhibited diminished prediction quality, resulting in imprecise cell boarders after the watershed. We retrained this model on data from multiple plates using the initial cell segmentation results as pseudo-ground-truth. Despite imperfections in these labels, the resulting network showed clearly improved predictions, generating cell segmentation results that were visually deemed of sufficient quality.

Cell classification was performed with a ResNet34 [32] that was applied to the barcode (mScarlet) channel, the Nucleus channel and the binarized cell segmentation mask centered around each cell and resized to the median cell size. The signal outside of the segmentation mask was removed to avoid crosstalk from the signal of other cells overlapping the bounding box of the current cell. This network was initially trained on several wells that contained isolated cell lines so that the expressed barcode was known for each cell without the need for human labeling. However, this network showed some inaccuracies when applied to images containing mixtures of barcodes. Consequently, we manually annotated 22 additional images by correcting the classification predictions of the initial network with MoBIE [33]. The network was further trained, and its accuracy was evaluated on separate splits of these data.

Initially, images were subjected to manual quality control to remove images with acquisition problems, such as out-of-focus images. The camera background was removed and spectral bleed-through correction was applied prior to cell segmentation and classification. Quality control was performed before obtaining the vp-score and Pearson’s correlation. The quality control consisted of the following steps:

1. Excluding images with less than 35 or more than 600 cells.
2. Removing 25% of cells of each cell class with the lowest intensity in spike and nucleocapsid expression in the Antigen expression channel.
3. Removing the cells that were classified as mScarlet-Lamin (non-expressing for viral protein) but showed an expression profile in the “Antigen expression” channel.
4. Removing the cells classified as spike-expressing cells (wt, delta, omicron) with a higher Antigen-expression value than 5000.
5. Removing the cells with a very small or large spatial size that likely correspond to segmentation errors.
6. Removing the cells with low expression in the barcode (mScarlet) channel defined by absolute intensity values for each cell line.
7. Removing the cells with a saturated expression level in the H2A Barcode channel.

Antibody binding was quantified based on immunofluorescence signal intensity in the IgG channel within the labeled masks. Signal intensity was measured in each pixel within the labeled mask to calculate the median signal intensity per cell. From this, median fluorescence intensity values across all cells expressing the same viral protein were calculated. The specific (above background) antibody binding (called “vp-score”) was calculated as the ratio between median fluorescence intensity in IgG channels of viral protein-expressing cells and cells expressing solely mSc-Lamin.

$$vp-score=\frac{Median\;intensity\;IgG\;(viral\;protein)}{Median\;intensity\;IgG\;(no\;viral\;protein)}$$

The vp-score for spike ≥ 1.3 and the vp-score for nucleocapsid ≥ 1.5, together with Pearson’s correlation ≥ 0.6, were empirically determined and considered indicative of specific binding in experiments with human sera. To compare the relative binding to different spike variants, we normalized the vp-scores to expression levels of each variant determined in the “Antigen expression channel”. The raw image data and all analysis results (cell segmentation, cell classification, quality control and further measurements) were visualized within the Fiji plugin MoBIE v4.2.3, enabling further visual quality control and an interpretation of the analysis results. The image analysis pipeline is available as open-source software at https://github.com/kreshuklab/covid-if-2 (accessed on 4 August 2024).

### 2.11. Statistical Analysis

Statistical tests were performed using Prism v5.01 (GraphPad Software Inc., Boston, MA, USA). Data were plotted using Prism v5.01 or the Python statistical data visualization package matplotlib v3.7.0, seaborn v0.12.2 and pandas v1.5.3. Median intensities were consistently used as the basis for all intensity measurements. Specific methodologies, including statistical tests and calculations, are provided in the figure legends.

## 3. Results

### 3.1. Setup of the Multiplex Microscopy Serology Assay

**Figure 1:**
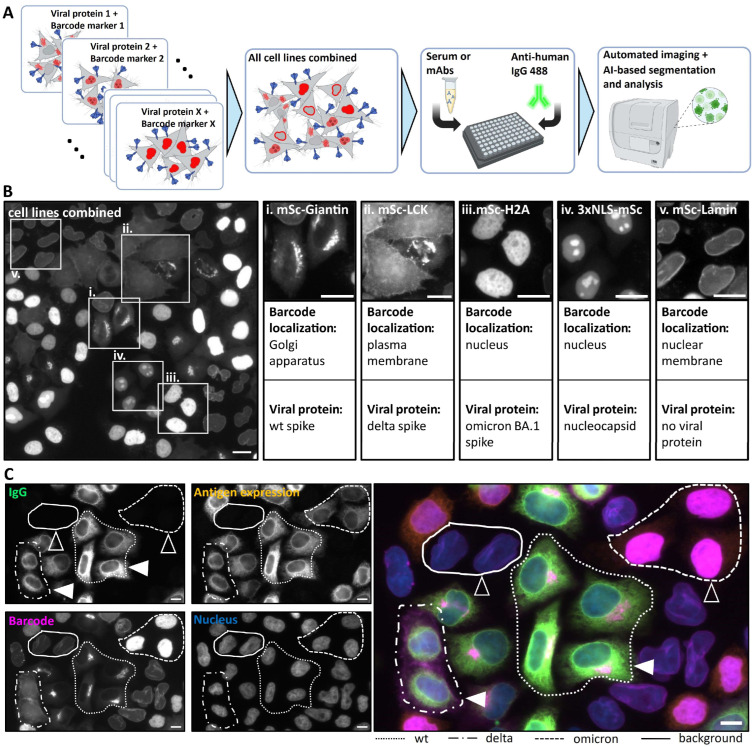
Principle of the multiplex microscopy serology assay. (**A**) Scheme of the multiplex assay workflow. Red structures represent fluorescent barcode proteins at different intracellular localizations, blue arrowhead-shaped structures represent viral proteins. (**B**) Cell lines transduced with different barcoding constructs and corresponding viral proteins were pooled and an overview image in the mScarlet channel was acquired. White boxes labeled with roman numbers mark the magnified areas showing cells with different subcellular localization patterns. Barcode localization and the corresponding viral protein are indicated below each image. (**C**) Images show results obtained using REGEN-COV therapeutic Ab on combined cell lines. Individual channels —“IgG channel” (represents REGEN-COV Ab binding, green), “Barcode channel” (represents viral antigen identity, magenta), “Antigen expression channel” (obtained by pan-specific anti-spike antibody staining and represents the expression levels of different spike variants, orange), “Nucleus channel” (obtained by Hoechst staining, blue) and an overlay image are shown. Cells expressing different variants are marked with different dashed line patterns (shown at the bottom of the image panel). Filled arrowheads indicate cells where REGEN-COV mAb binding can be detected, the empty arrowheads indicate those lacking detectable mAb binding. Scale bar = 10 µm.

A multiplex microscopy immunofluorescence assay was designed to simultaneously measure SARS-CoV-2 antibodies binding to a panel of viral proteins. This was achieved by generating a panel of cell lines, each engineered to express a different viral protein, and combining these cell lines in a single well for multiplex immunofluorescence analysis (Figure 1A). To allow for the identification of individual cell lines in the mixed population, we devised a barcoding strategy using mScarlet fluorescent protein targeted to specific subcellular compartments. Each viral protein variant expressed was paired with a unique barcode identifier (Figure 1A). Since barcodes are defined by localization patterns rather than by color, a single fluorescence channel is sufficient for the classification of multiple cell lines, each expressing a distinct viral protein.

To select fluorescent barcode markers that allowed robust discrimination between different cell lines, we screened a panel of mScarlet-tagged peptides and proteins with known subcellular localization [34] (Figure S2A). The criteria for suitability in our multiplex assay were the specificity of characteristic subcellular localization and stability of the distribution pattern upon fixation. We transfected HeLa cells with individually tagged mScarlet expression plasmids and imaged the cells before and after fixation (Figure S2B). Proteins targeting actin (LifeAct-mSc), microtubules (mSc-MapTau), focal adhesions (mSc-PXN), and peroxisomes (mSc-peroxisome) exhibited diffuse cytosolic localization after fixation and were deemed unsuitable for our assay (Figure S2B). The proteins mSc-Giantin (Golgi), LCK-mSc (plasma membrane), mSc-Lamin (nuclear envelope), 3xNLS-mSc (nucleus), H2A-mSc (nucleus), 4xmts-mSc (mitochondria), ER-mSc (endoplasmic reticulum) and Lamp1-mSc (Lysosomes) displayed characteristic localization patterns well retained upon fixation and were thus considered suitable for machine learning-based automated classification in our multiplex assay (Figure S2B).

Based on these results, we generated a panel of stably transfected HeLa cell lines, each expressing an mScarlet-tagged peptide or protein serving as a distinct subcellular barcode (Figure 1B). These were subsequently paired with the specific SARS-CoV-2 protein or protein variant. Specifically, we chose three variants of the spike (S) protein representative of different phases of the pandemic: Wuhan-Hu-1 (wild type), B.1.617.2 (delta) and BA.1 (omicron)). A Δ18-C-terminal truncated version of the spike protein that has enhanced expression efficiency but preserves the immunogenic potential of the wild type was used [35,36]. Additionally, the conserved nucleocapsid (N) protein, highly immunogenic and commonly used in diagnostic tests to indicate past SARS-CoV-2 infection across circulating variants [37,38], was included in our analysis. As an internal control for nonspecific staining, we employed a cell line expressing a subcellular localization marker (mScarlet-Lamin) without any SARS-CoV-2 antigen (Figure 1B). Cell lines were generated through sequential lentiviral-based transduction. The generated polyclonal cell lines underwent double Fluorescence-Activated Cell Sorting (FACS) to ensure the presence and appropriate expression levels of both proteins in each cell population (Figure S3).

To assess the assay’s ability to detect differential antibody binding, we employed a therapeutic mAb cocktail, REGEN-COV. This FDA-approved therapeutic comprises two non-competing human receptor-binding domain (RBD)-specific IgG antibodies—REGN10933 (Casirivimab) and REGN10987 (Imdevimab) [39,40]. Previous studies have demonstrated that these antibodies effectively bind and neutralize wild-type and delta variants of the spike protein, while proving ineffective against the omicron variant [14,41], rendering REGEN-COV an ideal candidate for our proof-of-principle experiments. The appropriate combination of barcoded HeLa cell lines was incubated with the mAb cocktail. Consistent with previous studies, mAb binding was only apparent on cells expressing wild-type and delta spike protein variants, identified by the expression of mSc-Giantin (Golgi) and LCK-mSc (plasma membrane), respectively (Figure 1C). In contrast, no binding was observed on cells expressing the omicron variant (barcoded by mSc-H2A signal in the nucleus) or those devoid of viral protein expression (barcoded by mSc-Lamin signal at the nuclear lamina) (Figure 1C). To ensure that all cells indeed express the viral variants and that the absence of omicron detection was not due to the lack of omicron variant expression, we stained the sample with a pan-specific anti-SARS-CoV-2 spike antibody (clone 1A9, Biozol (Eching, Germany) Cat.: GTX632604), targeting the conserved region (amino acids 1029–1192 of S2 subunit) of the spike protein. This confirmed the expression of all respective viral proteins (“Antigen expression”, Figure 1C). These results corroborate prior findings about the REGEN-COV binding across different SARS-CoV-2 variants [14,42,43], validating the assay’s capacity to detect differential antibody binding.

### 3.2. Machine Learning-Based Image Analysis Pipeline

**Figure 2:**
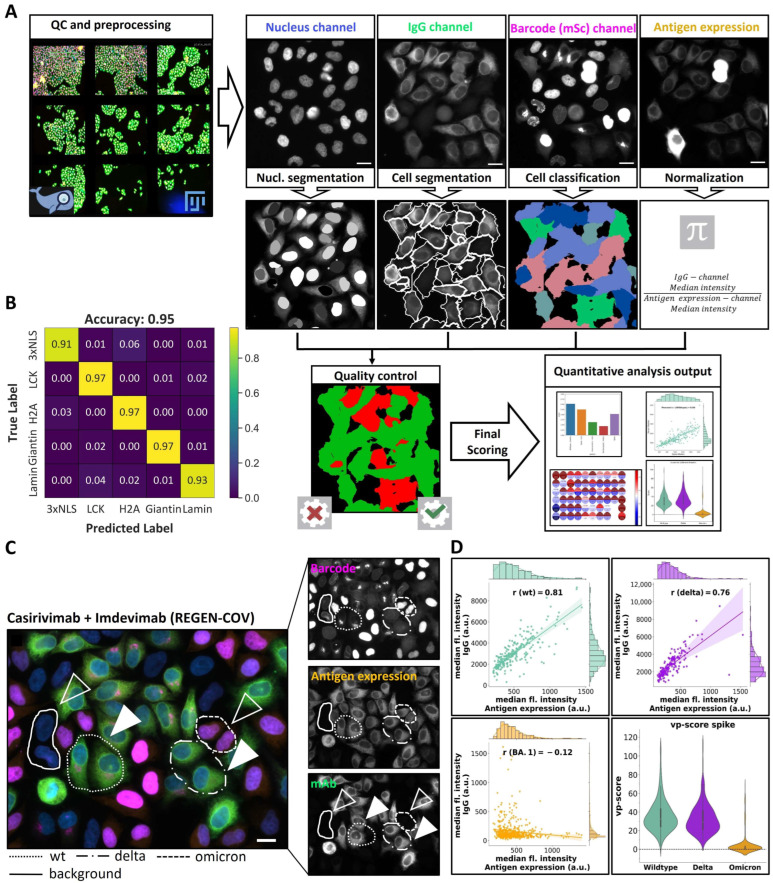
Image analysis and quantification pipeline. (**A**) Schematic overview of the image segmentation and classification workflow. A visual inspection of raw data (optional) and analysis results was conducted using FIJI [44] and MoBIE [33] software. Four channels were used for indicated purposes, and a final automated quality control was applied as described in the Materials and Methods section before computing the final scores. (**B**) Performance of the cell classification machine-learning model measured by confusion matrix showing the true cellular identity (True Label (manually classified), vertical) compared to predicted cellular identity (Predicted Label, horizontal). Color code (shown on the right) represents the fraction of classified cells in an indicated category. (**C**) Images show results obtained using REGEN-COV therapeutic Ab on pooled cell lines. Individual channels (“IgG” (green), “Barcode (mScarlet)” (magenta) and “Antigen expression” (orange)) and overlay are shown. Cells expressing different variants are marked with different dashed line patterns (shown at the bottom of the image panel). Filled arrowheads indicate cells where REGEN-COV mAb binding can be detected, and empty arrowheads indicate cells lacking detectable mAb binding. Scale bar = 10 µm. (**D**) Quantification of REGEN-COV mAb binding using the pipeline shown in A. Graphs show a correlation analysis for wild-type (green), delta (magenta) and omicron BA.1 (orange) spike variants between mAb binding quantified by median fluorescence intensity in “IgG channel” per cell and antigen expression quantified by median fluorescence intensity in “Antigen expression channel” per cell. Histograms on the vertical and horizontal axis represent the frequency of cells with indicated intensity values. Pearson correlation coefficient (r) is indicated within the plot area. Violin plots on the bottom right illustrate vp-score distributions for wt, delta, and omicron (BA.1) variants, where the shape of each violin represents the distribution of vp-scores, and the white dot within each plot indicates median vp-score.

To quantify results and facilitate high-throughput processing, we developed a fully automated analysis pipeline incorporating machine learning techniques. The analysis employed images recorded in four spectral channels captured using automated widefield microscopy—the “Nucleus channel” (for labeling nuclei, essential for quality control and cell segmentation), the “IgG channel” (for assessing specific antibody binding to various viral proteins), the “Barcode (mScarlet) channel” (for cell identification), and the “Antigen expression channel” (to control for viral protein expression levels using pan-specific spike antibody and/or nucleocapsid antibody) (Figure 2A).

Cell segmentation was achieved by utilizing a machine learning model established in our previous work [25], employing a seeded watershed algorithm. It uses the nuclei segmented with StarDist [30], applied to the nucleus channel as seeds, and boundary predictions from a U-Net [31], applied to the IgG channel as a heightmap. This segmentation method routinely produced boundaries matching those observed visually in raw microscopy images in the IgG channel (Figure 2A).

Subsequently, we introduced a novel approach that automatically classified cells based on the expressed fluorescent markers with distinct subcellular localization. The approach relies on a deep neural network for supervised image classification (ResNet 34 [32]) (Figure 2A), applied to the image data of each segmented cell individually. The classification model was trained as described in the Materials and Methods section. To validate the effectiveness of our barcoding strategy, we applied the trained model to a new dataset that has not been used for training (unseen sample) comprising a mixture of cell lines expressing different barcodes. Remarkably, the model achieved an accuracy of 95.5% (Figure 2B). Notably, our model reliably distinguished the three different nuclear markers used in our setup (mSc-Lamin, mSc-H2A and 3xNLS-mSc) based on relatively subtle differences in nuclear staining patterns. 

In summary, our approach combines machine learning-based segmentation and classification to automatically generate cell masks and categorize them into five classes based on the expressed viral protein. Potential unspecific antibody binding to cell surfaces was determined based on the IgG channel intensity of mSc-Lamin cells, which do not express any viral proteins. Antibody-binding was calculated as the ratio between median fluorescence intensity in IgG channels of viral protein-expressing cells and cells expressing solely mSc-Lamin (called viral protein (vp-score)), as described in the Materials and Methods section.

To further assess binding specificity, we obtained intensity measurements of viral protein expression from the “Antigen expression channel”. It would be expected that specific antibody binding (IgG channel) is proportional to viral protein expression. Therefore, we calculated the Pearson’s correlation coefficient (r) between these two channels.

To validate our analysis strategy, we applied the automated pipeline to the REGEN-COV mAb experiment described in Figure 1, which was previously only qualitatively evaluated. The derived quantitative data revealed negligible binding to the omicron variant (vp of 1.24 and r of −0.12) compared to binding to the wild type and delta spike variants, evidenced by high vp-score and Pearson’s correlation coefficient (r) values (vp-score of 32.4 and 29.1, respectively; r of 0.81 and 0.76, respectively (Figure 2C,D)). To assess the sensitivity and dynamic range of the assay, we performed a titration experiment using the REGEN-COV mAb cocktail. mAb binding above the background was detectable for wild-type and delta variants at concentrations as low as 4 ng/mL of each mAb, reaching a plateau at around 200 ng/mL (Figure S4A). Notably, REGEN-COV comprises two distinct therapeutic mAbs—Casirivimab and Imdevimab. In individual dilution series experiments, Casirivimab exhibited stronger binding to the wild-type and delta spike variants compared to Imdevimab, with neither antibody demonstrating significant binding to the omicron variant (Figure S4B). Since Casirivimab and Imdevimab target different spike epitopes and can bind non-competitively [39], we investigated whether this was reflected in our assay. Cells were exposed to saturating concentrations of Casirivimab (1.8 μg/mL), Imdevimab (1.8 μg/mL), and a combination of both mAbs (where each mAb is present at half the concentration compared to the individual samples), and spike protein binding was assessed. The combination of both antibodies resulted in higher IgG channel fluorescence intensity measurements compared to individual mAb treatments (Figure S4C). This highlights the assay’s capability to detect binding to distinct epitopes on the spike protein and the potential synergistic effects.

In summary, our proof-of-concept experiments using the REGEN-COV therapeutic mAb cocktail successfully validated the assay’s capacity to quantitatively assess differential antibody binding to distinct viral proteins with high sensitivity as well as antibody binding to different epitopes on the same viral protein.

### 3.3. Testing of Monoclonal Antibodies against SARS-CoV-2 Spike Protein

**Figure 3:**
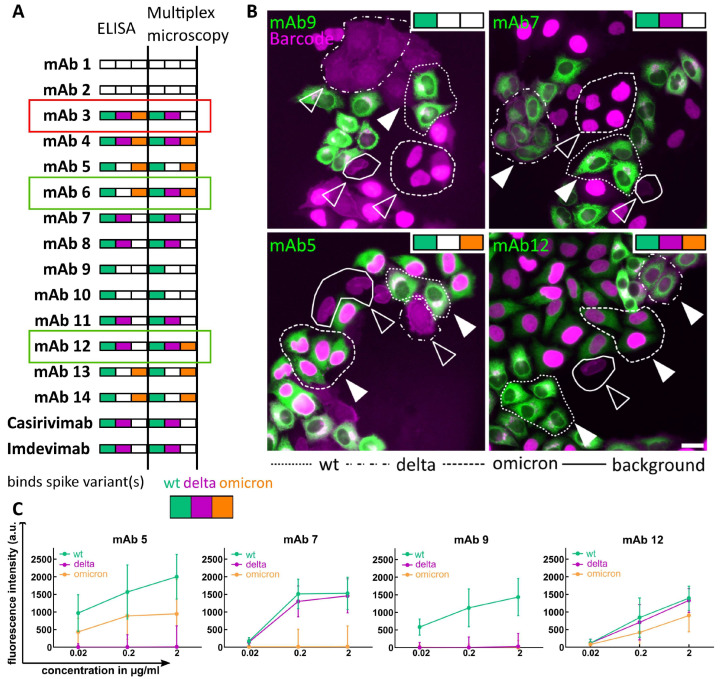
Assessment of mAb binding to different spike variants. (**A**) Qualitative comparison of differential mAb binding to different spike variants between ELISA (data from Wang et al. [29]) and the multiplex microscopy approach. Colored boxes (green (wt), magenta (delta) and orange (omicron)) indicate detected binding; white box: no binding. mAbs exhibiting some discrepancy between methods are marked with red (microscopy assay not detecting variant binding) and green (those where additional variant binding was detected by microscopy) boxes. Binding was evaluated as positive if median fluorescence intensity in the “IgG channel” was above 150 and more than three times higher than Casirivimab and Imdevimab binding to the omicron variant (negative control). (**B**) Representative images showing overlay of the indicated mAb and Barcode channels, illustrating differential spike binding specificity. Color bar in the top left corner indicates binding to the respective variant, and the gray box indicates no binding. Filled arrowheads: mAb binding detected; empty arrowheads: cells lacking detectable mAb binding. Scale bar = 10 µm. (**C**) Examples of quantitative data derived from results shown in B. Background-subtracted median fluorescence intensities are plotted against mAb concentration. Error bars show the standard deviation of median intensity values.

Building on the results described above, we tested the capacity of our assay to characterize a comprehensive set of mAbs. For this, we obtained a panel of 14 mAbs with known specificity for wild-type, delta and omicron spike variants [29]. We selected mAbs, which exhibited differential binding patterns to the respective spike variants, as shown in Figure 3A (binders with EC50 < 1000 ng/mL are indicated with variant-specific colors).

The multiplex microscopy assay was employed (Figure 1A) together with the automated machine learning-based analysis pipeline (Figure 2A) to quantify the binding of each of the 14 mAbs at three different concentrations to the wt, delta and omicron spike variants. This yielded a total of 42 antibody–epitope combinations that were evaluated and compared to published data from Wang et al. [29]. Our analyses revealed differential binding of 14 mAbs across different spike mutants in a concentration-dependent manner (Figure 3A–C and Figure S5A). The specificity of the spike epitope binding determined here closely matched that previously reported by Wang et al. in almost all cases (39/42, Figure 3A). In two cases (mAb 6 and 12), the assay used here revealed, in addition to wild type, binding to a delta or omicron variant, respectively, which was not detected by ELISA measurements in Wang et al. (Figure 3A).

In the case of mAb 3, our assay failed to detect the binding to the omicron BA.1 variant (Figure 3A and Figure S5A) found by ELISA in Wang et al. To test whether PFA fixation prior to epitope staining had compromised the detection of certain epitopes, we performed antibody staining before PFA fixation. Indeed, this live cell labeling approach rescued the binding of mAb 3 to the omicron BA. 1 variant (Figure S5B). 

In conclusion, the multiplex microscopy serology assay demonstrated the capacity to detect the differential binding of mAbs across various spike variants in very good agreement with the ELISA-based measurements, verifying the assay’s potential to be used for mAb screening approaches.

### 3.4. Screening of Human Sera

Next, we investigated the diagnostic utility of the assay for serological characterization of human samples. Therefore, we added a cell line expressing the SARS-CoV-2 nucleocapsid protein to our panel. The presence of the anti-nucleocapsid antibodies is indicative of exposure to SARS-CoV-2 and serves as a differentiator between vaccinated and acutely infected or convalescent individuals. To determine the specificity and sensitivity of the assay, we employed the positive and negative control cohorts established in our previous work [25]. The negative control cohort (n = 233) consisted of sera samples collected from healthy individuals, from patients infected with common cold coronaviruses (ccCoV), as well as patients infected with Mycoplasma pneumonia, Epstein–Barr virus (EBV) and Cytomegalovirus (CMV), all collected before 2019. The SARS-CoV-2 (positive control) cohort comprised 65 serum samples from 41 acutely infected patients confirmed positive for SARS-CoV-2 by RT-PCR in 2020 and collected between day 2 and 29 post-infection [25]. All samples were analyzed by our multiplex microscopy assay, the vp-score was calculated for spike (represented as an average vp-score across all three spike variants tested) and nucleocapsid proteins and compared to results obtained by a commercially available semi-quantitative SARS-CoV-2 ELISA (Euroimmun, Lübeck, Germany) approved for diagnostic use for the presence of SARS-CoV-2 spike-specific IgG.

**Figure 4:**
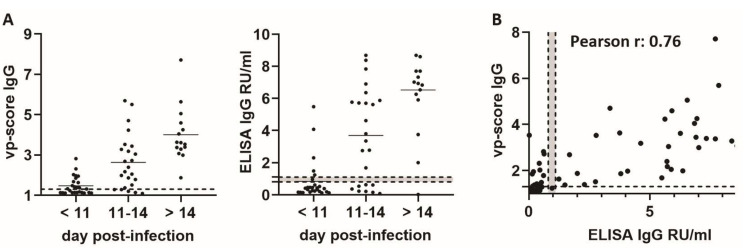
Comparison of multiplex microscopy assay and ELISA in the detection of SARS-CoV-2 spike antibodies in patient sera. (**A**) Samples from the positive control cohort have been stratified into three groups based on the day post-infection and analyzed by multiplex microscopy assay (**left**) and ELISA (**right**). Each dot represents one serum sample. Line = mean value; (**B**) correlation between vp-score obtained by multiplex microscopy assay and ELISA measurements; dotted line at 1.3 for vp-score = empirically determined cut-off value used to classify sera as negative or positive; dotted lines with gray area between 0.8 and 1.1 RU/mL in ELISA plots represent borderline situations where sera cannot be classified as positive or negative according to the manufacturer’s protocol.

In the negative cohort, 3 out of 233 samples (1.3%) had a false positive vp-score (vp > 1.3) for anti-spike, while 3 had a false positive vp-score (vp > 1.5) for anti-nucleocapsid without any overlap between the spike- and nucleocapsid-positive samples. A similar proportion (n = 7, 3%) scored false positive in ELISA measurements (Table 1, Figure 4A), but there was no overlap of false positive samples between our assay and ELISA. To evaluate the results from the positive cohort, we stratified the samples into three groups based on the day of sample collection post-infection (<11 days (n = 26); 11–14 days (n = 24); >14 days (n = 15)). The stratification was based on previous research on SARS-CoV-2 IgG seroconversion after first infection, where seroconversion for IgG targeting the spike protein was detected between day 10 and 14 post-infection with ELISA and CLIA [45,46,47]. All (15/15) of the samples collected >14 days post-infection exhibited a positive vp-score for both spike and nucleocapsid, compared to 14/15 (93.3%) in the ELISA assay. In the “11–14 days” and “<11 days” groups, 19/24 (79.1%) and 14/26 (54%) scored positive in our assay compared to 16/24 (66.7%) and 6/26 (23.1%) determined by ELISA (Table 1, Figure 4A). In general, the vp-score values correlated well with the ELISA scores (Pearson correlation r = 0.76) (Figure 4B). In summary, the results demonstrate that the multiplex microscopy serology assay can provide semi-quantitative measurements of spike and nucleocapsid reactive antibodies in human sera with specificity and sensitivity similar or better than an ELISA assay approved for clinical diagnostic use.

**Figure 5:**
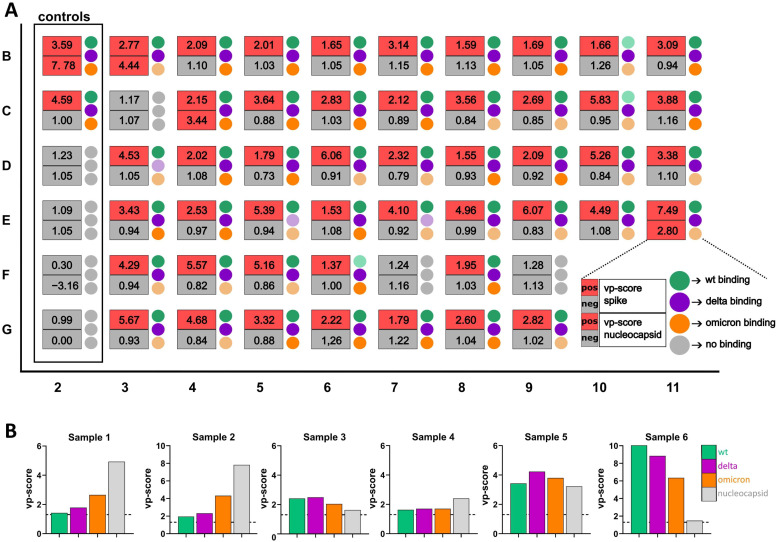
Analysis of the serological status of vaccinated and omicron-infected individuals. (**A**) Exemplary data showing the multiplex microscopy results of sera obtained from 50 vaccinees in the multi-well plate. The cells in the first column (2) are used as controls and are incubated with sera obtained from SARS-CoV-2-infected and -vaccinated individuals (wells B2 and C2, positive controls), pre-pandemic sera (wells D2 and E2, negative controls) and no sera (wells F2 and G2, background and bleed through control wells). The numbers in each box show the vp-score for spike binding (upper number, red = positive (vp > 1.3), gray = negative) and nucleocapsid binding (lower number, red = positive (vp > 1.5), gray = negative. The colored circles on the side of each box represent binding to different spike variants (green (wt), magenta (delta), orange (omicron) and gray (no binding). Dim color represents at least 15% weaker binding to the indicated variant compared to the variant where the strongest binding was measured. (**B**) Bar graphs show the vp-scores for different spike variants (green (wt), magenta (delta), orange (omicron)) and the nucleocapsid (gray) of omicron-infected individuals. Dotted line at 1.3 for vp-score = empirically determined cut-off value used to classify sera as negative or positive.

Finally, we employed the assay to analyze the serological status of vaccinated individuals. This is particularly relevant in the context of a potential need for recurring vaccinations. For this, we examined sera from a cohort of n = 50 individuals who had received two doses of the Pfizer-BioNTech BNT162b2 vaccine in early 2021 at least 14 days before sample collection (Figure 5A). Vaccinees had not experienced a prior SARS-CoV-2 infection according to their own statements. Of the 50 tested sera, 47 scored positive for spike antibodies in our assay (Figure 5A), indicating that two doses of vaccine had resulted in the generation of reactive antibodies. Notably, the three samples (wells C3, F7 and F9, Figure 5A) that scored negative in this assay were also negative when tested with ELISA measuring anti-spike IgG (scoring 0.98, 0 and 0.03 RU/mL, respectively, with a cutoff for positivity being > 1.1 RU/mL), indicating low response to the vaccine. The vp-score for nucleocapsid was negative for 47 of 50 samples. Only three sera exhibited positive reactivity to the nucleocapsid protein in both IF assay and ELISA (wells B3, C4 and E11), indicating prior infection with SARS-CoV-2. 

We also examined differences in antibody binding to the different spike variants in the vaccinee cohort (Figure 5A). Reactivity of the sera to all three spike variants was detected, indicating the presence of antibodies targeting conserved epitopes. However, in the majority of vaccinee samples, we detected more antibody binding to wild-type and delta spike proteins, while binding to the omicron variant was often weaker. This pattern of differential binding is not surprising considering that the BNT162b2 vaccine is based on the wild-type spike with the delta variant being relatively similar (8 mutations in the spike protein) and omicron considerably more divergent (33 mutations in the spike protein) [16,48].

To further investigate the effectiveness of a semi-quantitative assessment of antibody binding affinity across various spike variants and apply it in a more complex clinical situation, we analyzed a small cohort of six patients, where sequencing confirmed acute SARS-CoV-2 infection with the omicron variant (Figure 5B). The samples were collected at least 15 days post-infection except for sample 3 (8 days post-infection). Five of these individuals were unvaccinated before the omicron infection (samples 1–5), while one was vaccinated with the BNT162b2 vaccine prior to the omicron breakthrough infection (sample 6). 

For two out of the five individuals who were unvaccinated, we detected antibodies that preferentially targeted the omicron variant while three sera displayed similar binding across all variants (Figure 5B). For the vaccinee who experienced an omicron breakthrough infection, we measured the highest vp-score for the wild-type variant, which is in line with reports indicating reactivation of preexisting cross-reactive B cells generated following breakthrough infection with a different strain [11,29,49,50] (Figure 5B, sample 5).

In summary, the multiplex microscopy serology assay was successfully employed in the semi-quantitative characterization of human samples in the context of determining the effectiveness of the vaccination procedure, previous (unknown) exposure to SARS-CoV-2 and disentangling preferential antibody binding across different spike variants, providing information relevant in situations of continued emergence of new virus variants as well as in clinical settings.

## 4. Discussion

We developed and validated a microscopy-based, multiplex serology assay, for the simultaneous detection and characterization of pathogen-specific antibodies. The assay integrates automated high-resolution microscopy in multi-well plates (Figure 1) and a robust machine learning analysis pipeline enabling a fully automated workflow (Figure 2). Serological assays like ELISA and (E)CLIA are a gold standard in antibody detection following viral infections [51]. However, the microscopy-based approach presented here has several unique aspects that may prove advantageous under certain conditions. Standard serological assays mainly employ purified recombinant proteins as antigens [20,52]. Potential complications may arise when proteins are expressed and purified under conditions that do not represent their native environment, compromising the attainment of biochemical properties essential for viral tropism and antigen specificity, such as folding and glycosylation patterns [20]. In addition, some proteins, in particular membrane proteins, are challenging to express and purify in large quantities *in vitro*. In the cell-based assay presented here, the protein of interest is expressed, processed and transported in a near-native cellular environment. This should facilitate the detection of antibodies that recognize the native conformation. The assay could be performed either with or without permeabilization. Here, we employed permeabilization since we were aiming for a parallel detection of intracellular and extracellular epitopes as well as to describe conditions that are broadly applicable for other viral antigens, independent of intracellular localization. Differential fixation can also reveal epitopes with different levels of glycosylation for comparison. The assay developed here, together with the panel of barcoded cell lines, represents a streamlined, rapidly adaptable, biosafety level 1-compatible toolbox that primarily requires the sequence of the protein of interest for cloning into a lentiviral vector. While some optimization of expression might be necessary when adapting the assay to other viral antigens, this renders the assay useful in the context of emerging pathogens since it can be rapidly developed as soon as the genomic sequence of the pathogen and relevant open reading frames are known. 

A unique feature of the assay, distinguishing it from other microscopy-based serological assays described recently [25,53,54], is the multiplexing approach that uses fluorescent protein localization rather than its spectral characteristics as a barcoding strategy (Figure 1 and Figure S2). Together with machine learning-driven cell classification and analysis (Figure 2), our assay is able to identify and characterize the differential binding of antibodies to various viral epitopes and variants within one experiment. Pietiäinen et al. previously demonstrated multiplex serology assay testing for various viral SARS-CoV-2 proteins [53]. Although the assay by Pietiäinen et al. investigated binding to S, N and M proteins of SARS-CoV-2, the multiplex approach faced constraints due to limitations in the number of available spectral channels and had to employ separate wells for each viral antigen. Our multiplex strategy not only simplifies the experimental process but also enhances its efficiency by allowing the simultaneous analysis of multiple targets. The approach can be extended to a larger number of viral proteins by employing the additional barcoding markers identified in this study (e.g., those localizing to lysosomes, ER or mitochondria). Although the system is limited by the number of markers with unique subcellular localization, machine learning-based pattern recognition and classification can recognize even subtle differences in localization patterns and are constantly improving. This is demonstrated by the fact that three different nuclear markers (H2A, lamin and NLS) were accurately classified by machine learning based on relatively subtle staining pattern differences. In addition, combining two or more barcodes in one cell will dramatically increase the number of different subcellular localization patterns that can be used for cell classification. Thus, we believe that the number of barcode proteins suitable for this multiplexing approach can be substantially expanded. Since barcoding uses only one spectral channel, one can take advantage of other spectral channels to assess, e.g., the presence of IgM and IgA in parallel to IgG.

In this proof of principle study, we applied the assay to the most relevant use-case situations, e.g., the testing of mAbs with potential therapeutic application (Figure 3) and serological analysis of human sera (Figure 4 and Figure 5). Therapeutic mAbs represent a crucial treatment pillar in the early phase of pandemics and form an essential component of the portfolio of treatment options against newly emerging pathogens [55,56]. Our analysis of therapeutic mAb REGEN-COV and 14 mAbs from Wang et al. with known spike-variant binding specificity was largely in agreement with published data [29] (Figure 3 and Figure S5). The few cases where we detected a discrepancy between the multiplex microscopy assay and ELISA highlight the subtle differences between methods. Given these results, as well as the costs and risks involved in mAb development, we imagine the multiplex microscopy assay could be integrated into a large-scale, commercial mAb discovery pipeline as an alternative approach to validate positive hits obtained by ELISA. Here, a multiplex option and the capacity to detect synergistic binding could be exploited to generate cells expressing different segments of the immunodominant protein to test for specific antibody combinations.

The multiplexing microscopy approach is particularly relevant for serological analysis of human serum samples, which often contain a complex array of antibodies against various viral components, depending on the vaccination status and previous exposure to viral variants. The lack of overlap in false positives between ELISA and multiplex microscopy assay (Figure 4, Table 1) indicates the potential for leveraging a dual-readout strategy to achieve extremely accurate specificity in critical applications such as serological screening in low seroprevalence populations [26]. Our analysis of vaccinee and omicron-infected individuals revealed differential antibody binding to spike variants and nucleocapsids consistent with their vaccination status and medical history (Figure 5). The ability of the assay to provide a “serological fingerprint” of SARS-CoV-2 binding antibodies for each individual enables addressing the inherent variability in the antibody landscape. This is particularly relevant in the context of the extensive vaccination efforts and continuously evolving virus variants.

The main limitation of the approach is the requirement for expertise and infrastructure for cell culture, microscopy and image analysis. The assay is, therefore, not considered as an alternative to commercially available assays for routine diagnostics. However, it can present an important experimental alternative in the context of newly emerging viral pathogens when information about immuno-relevant epitopes has yet to be obtained and when well-established serological assays are not available or do not reflect rapid viral evolution in a pandemic scenario.

## 5. Conclusions

In conclusion, we developed a microscopy assay suitable for the detection of antibodies against multiple viral proteins simultaneously. For multiplexing, we used a barcoding strategy that relies on subcellular localization of mScarlet-tagged peptides/proteins enabling evaluation of antibody binding to a large number of targets by using only a single spectral channel. Together with automated microscopy and the developed machine learning-based image segmentation, classification and analysis, the assay is compatible with high throughput applications. We applied this approach for quantitation of antibody binding to different variants of SARS-CoV-2 spike and nucleocapsid proteins in sera of patients or vaccinees, as well as for the study of reactivity of monoclonal antibodies. Importantly, given the compatibility with biosafety level 1 and the potential for rapid adaptability to emerging pathogens, the assay represents an important resource in the context of pandemic preparedness and an alternative to standard serological assays based on purified proteins.

## Figures and Tables

**Table 1:** Summary of positive results for control samples obtained by ELISA and multiplex microscopy assay.

Cohort	Microscopy Assay Spike IgG	ELISA Spike IgG
Negative cohort n = 233	3/233 (1.3%)	7/233 (3%)
SARS-CoV-2 cohort < 11 days post-infection n = 26	14/26 (53.8%)	6/26 (23.1%)
SARS-CoV-2 cohort 11–14 days post-infection n = 24	19/24 (79.1%)	16/24 (66.7%)
SARS-CoV-2 cohort > 14 days post-infection n = 15	15/15 (100%)	14/15 (93.3%)

## Data Availability

The original contributions presented in the study are included in the article. Requests for reagents developed in this work such as cell lines and plasmids as well as raw images can be directed to the corresponding author. The image analysis pipeline is available as an open-source software at https://github.com/kreshuklab/covid-if-2 (accessed on 4 August 2024).

## Supplementary Materials

The following supporting information can be downloaded at: https://www.mdpi.com/article/10.3390/v16091473/s1, Figure S1: Schematic representation of plasmids generated for this study; Figure S2: Evaluation of subcellular localization of mScarlet-tagged peptides and proteins in living and fixed cells; Figure S3: Sorting of the cell lines; Figure S4: Quantification of the sensitivity and specificity of multiplex microscopy assay in mAb evaluation; Figure S5: mAb binding quantification; Table S1: List of plasmids used and generated in the study; Table S2: List of cell lines used or generated in the study; Table S3: List of used mAbs from Wang et al. and their binding specificities.
