# Supplementary material for: Multiplex Microscopy Assay for Assessment of Therapeutic and Serum Antibodies against Emerging Pathogens
Source: Viruses. 2024 Sep 17;16(9):1473. doi: 10.3390/v16091473 (PMC11437451; doi:10.3390/v16091473)
Supplement: Supplementary file 1 [file viruses-16-01473-s001.zip › viruses-3168511-Supplementary-published update.pdf]

Supplementary Material to article:

„Multiplex microscopy assay for assessment of therapeutic and serum antibodies against emerging pathogens"

Figure S1. Schematic representation of plasmids generated for this study.

Figure S2. Evaluation of subcellular localization of mScarlet-tagged peptides and proteins in living and fixed cells.

Figure S3. Sorting of the cell lines.

Figure S4. Quantification of the sensitivity and specificity of multiplex microscopy assay in mAb evaluation.

Figure S5. mAb binding quantification.

Table S1. List of plasmids used and generated in the study

Table S2. List of cell lines used or generated in the study

Table S3. List of used mAbs from Wang et al. [19] and their binding specificities

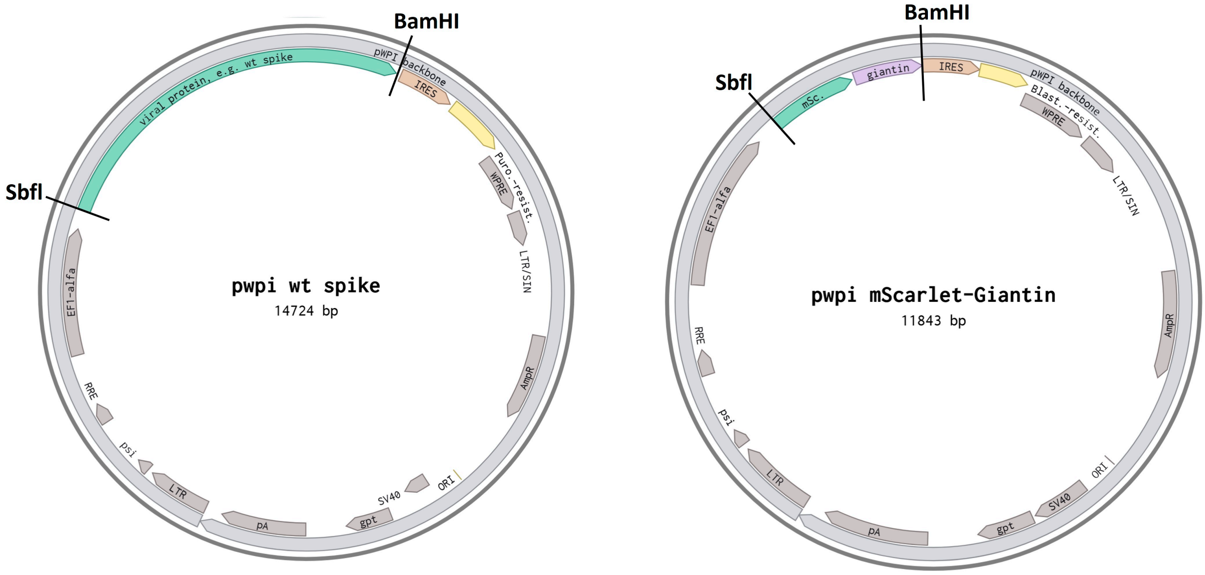

Figure S1. Schematic representation of plasmids generated for this study. left: vector expressing the viral protein (spike protein is shown as an example), right: vector expressing the barcode protein (mScarlet-Giantin is shown as an example).

A

Plasmid name	Addgene ID	Expected subcellular localization
pLifeAct_mScarlet-i_N1	85056	actin
pmScarlet-I_peroxisome_C1	85065	peroxisomes
mScarlet-I-PXN	112956	focal adhesions
mScarletl_MapTauC	112964	microtubules
4xmts-mScarlet-I	98818	mitochondria
ER-mScarletI	137805	endoplasmatic reticulum
Lamp1-mScarlet-I	98827	lysosomes
pmScarlet-i_H2A_C1	85053	nucleus
mScarlet-I-LaminB	98831	nuclear membrane
3xnls-mScarlet-I	98816	nucleus
pmScarlet-i_Giantin_C1	85050	Golgi apparatus
Lck-mScarlet-I	98821	plasma membrane

B

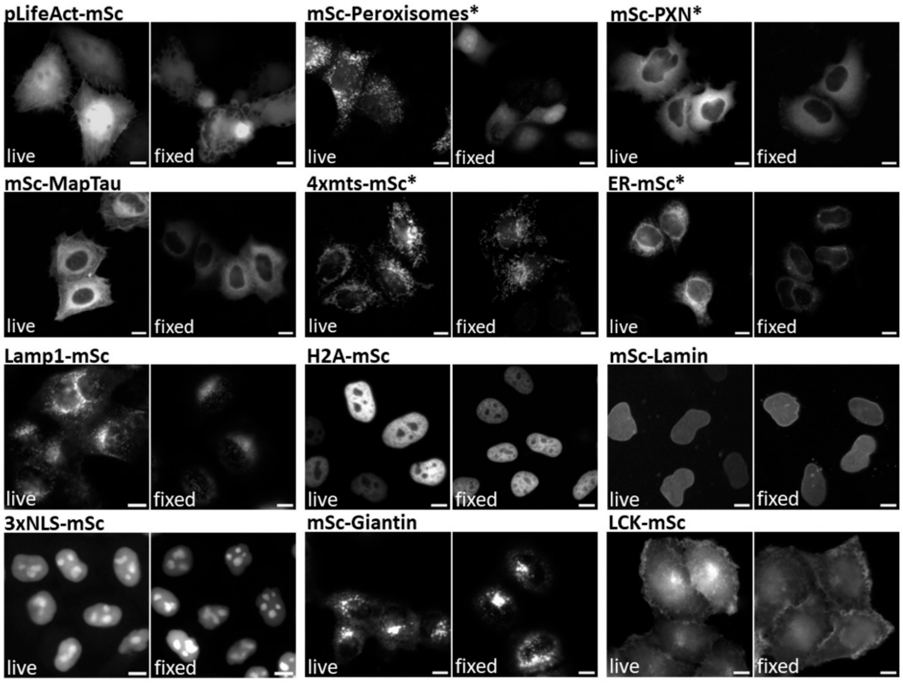

Figure S2. Evaluation of subcellular localization of mScarlet-tagged peptides and proteins in living and fixed cells. (**A**) List of mScarlet-tagged peptides and proteins with characteristic subcellular localization that were tested in this study (**B**) Fluorescence images of exemplary HeLa cells transfected with plasmids shown in A. At 24h post transfection cells were imaged in the mScarlet channel (“live” panels), fixed with 4% PFA for 20 min and imaged again (“fixed” panels). The same brightness and contrast display settings

for "live" and "fixed" samples were used within each sample. Samples marked with “*“ are shown with 30% increased brightness/contrast setting for “fixed” condition compared to “live” condition to facilitate visualization. Scale bars = 10 µm.

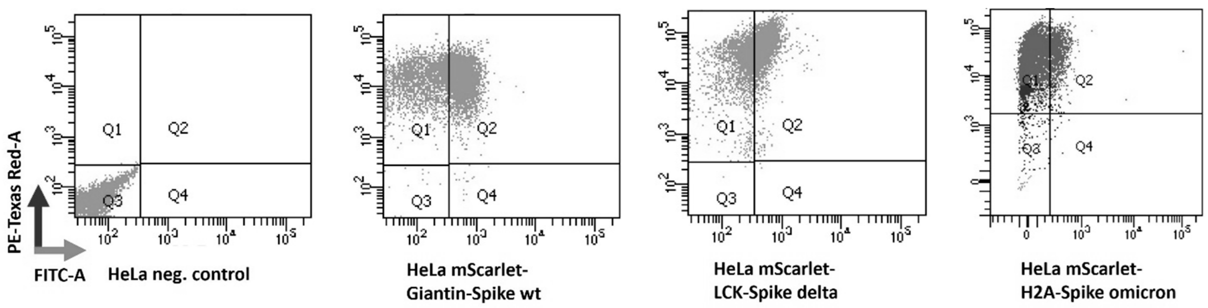

Figure S3. Sorting of the cell lines. FACS scatter plots (spike expression (x axis), barcode expression (y axis)), together with thresholds used for sorting the double-positive population (Q2) are shown. The names of the sorted cell lines are shown below.

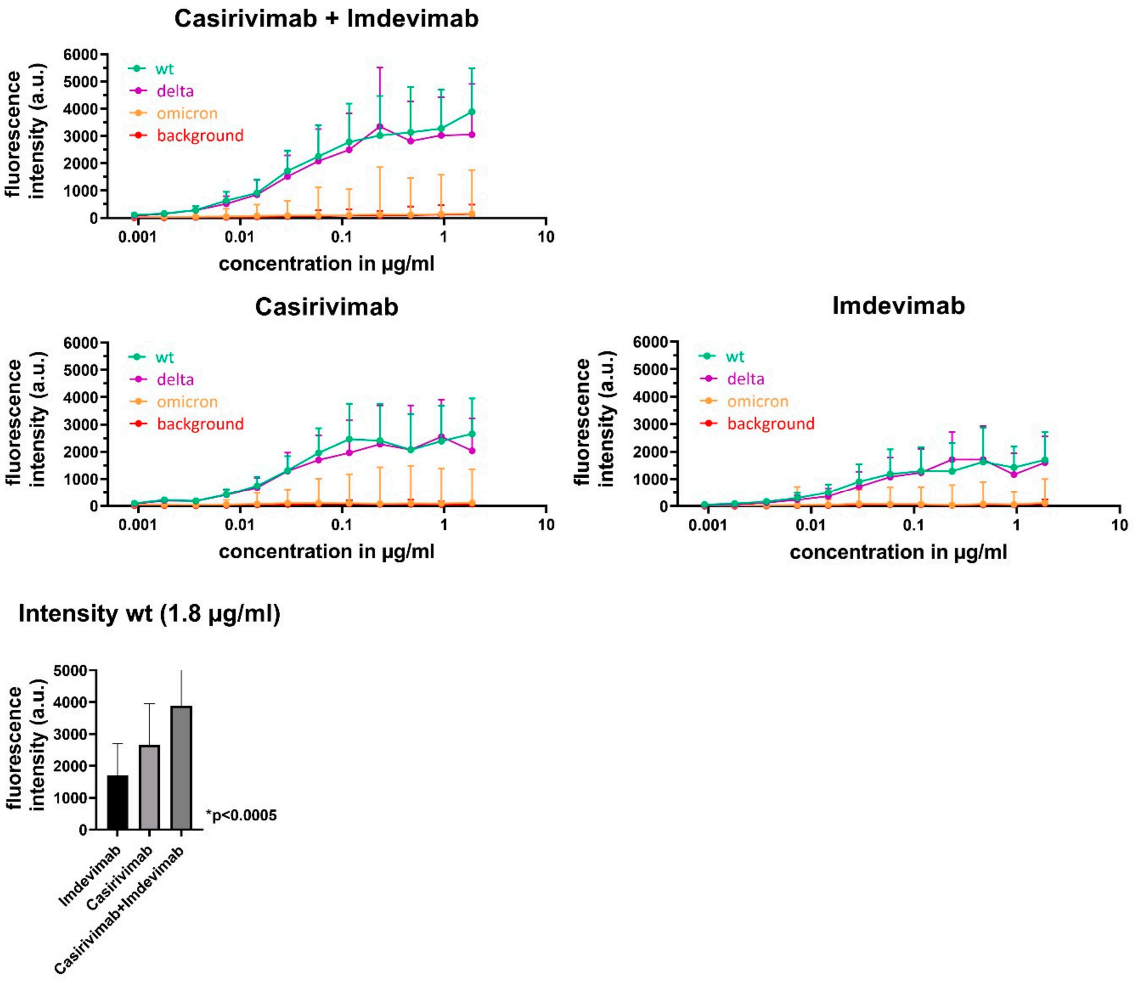

Figure S4. Assessment of the sensitivity and specificity of multiplex microscopy assay in mAb evaluation. **(A, B)** The binding of REGEN-COV therapeutic mAb cocktail (A) and of the individual mAbs (B) to different spike variants was assessed using the multiplex microscopy assay as described in Figures 1 and 2. The graph shows dependence of fluorescence intensity microscopy measurement (represented as median fluorescence intensity across all cells expressing the same spike variant) on concentration of REGEN-COV cocktail across different spike variants (wt (green), delta (magenta), omicron (orange)) and background (red). **(C)** The graph shows fluorescence intensity measurements for wt spike binding in response to treatment with individual mAbs (Imdevimab, Casirivimab) and both mAbs combined all at the final concentration of 1.8 µm/ml. Error bars represent standard deviation of the median fluorescence intensity measured across at least 200 cells. The effect on median fluorescence intensity signal of both mAbs combined was significantly higher than the effects of Casirivimab alone ($p = 0.000021$) and Imdevimab alone ($p = 0.000012$) assessed with a paired t-test. Asterisks (*) indicate statistically significant differences between the groups. A p-value < 0.05 was considered statistically significant

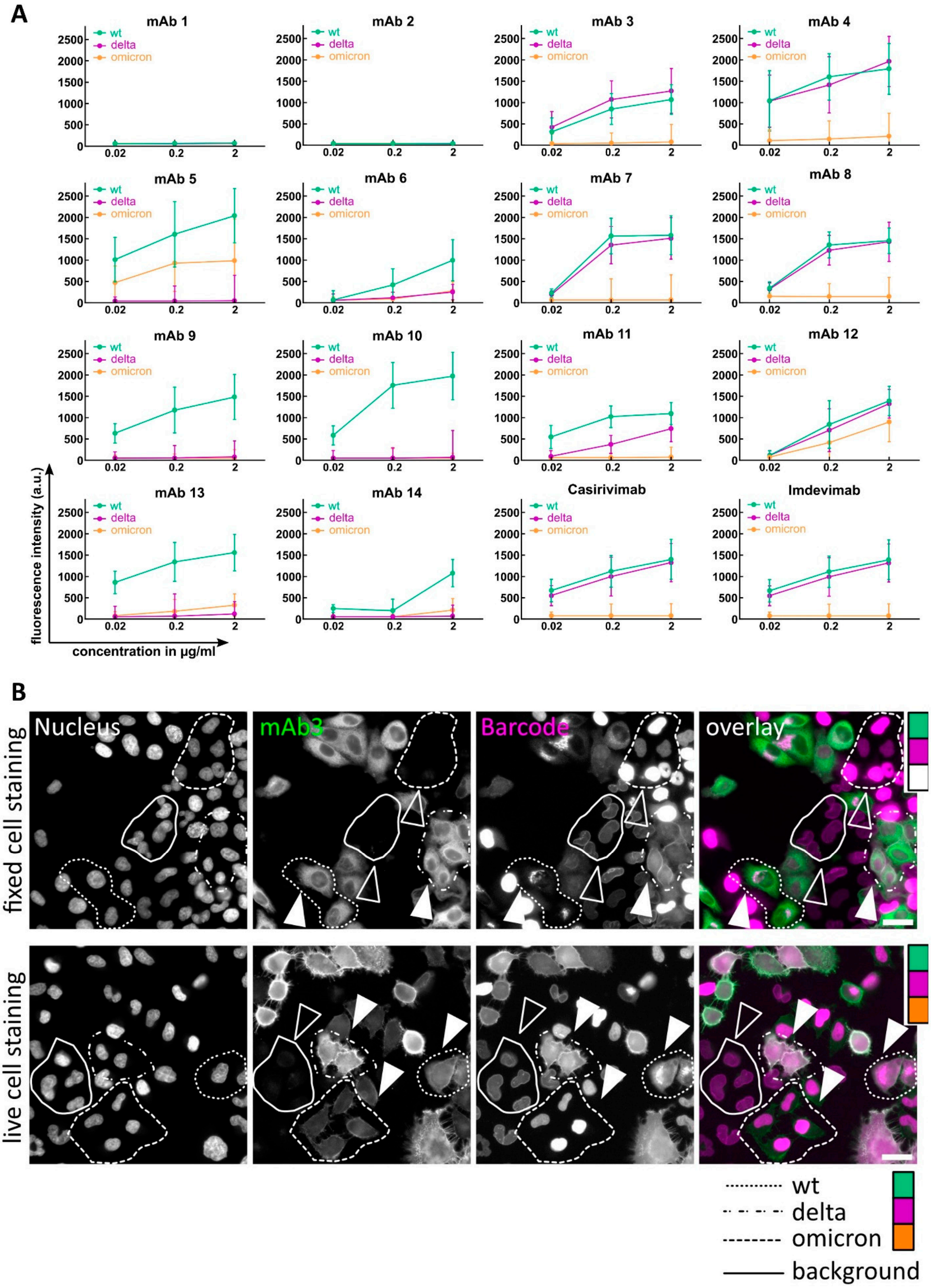

A
mAb 1
mAb 2
mAb 3
mAb 4
mAb 5
mAb 6
mAb 7
mAb 8
mAb 9
mAb 10
mAb 11
mAb 12
mAb 13
mAb 14
Casirivimab
Imdevimab
wt
delta
omicron
0
500
1000
1500
2000
2500
0.02
0.2
2
fluorescence intensity (a.u.)
concentration in µg/ml
B
Nucleus
mAb3
Barcode
overlay
fixed cell staining
live cell staining
wt
delta
omicron
background

Figure S5. Assessment of mAb binding to three different spike variants. (**A**) Three concentrations of all 14 mAbs were analyzed by the multiplex microscopy assay. Median fluorescence intensities of at least 200 measured cells are plotted against concentration of mAbs for each SARS-CoV-2-spike protein (indicated in colours – wt (green), delta (magenta) and omicron (orange)). Error bars show standard deviation of median intensity values. The identity of the tested mAb is shown above the graph. (**B**) Panels show binding of mAb3 before (top panel) and after fixation (lower panel). Individual channels ("mAb3" (green), "Barcode" (magenta) and "Nucleus") and an overlay of "mAb3" and "Barcode" channels are shown. Groups of cells expressing different variants are marked with different dashed line patterns (shown at the bottom of the image panel). Filled arrowheads indicate cells where mAb3 binding can be detected, empty arrowheads indicate cells lacking detectable mAb3 binding. The color scheme on the right side indicates the binding specificity of the mAb3 in different conditions where green, magenta and orange color indicate binding to the respective spike variant. White box = no binding. Scale bar = 10 µm.

Table S1. List of plasmids used and generated in the study

Parental Plasmids		
Name	Reference	Information
pWPI Puro	Trotard et al., 2016 [17]	Lentiviral transfer plasmid containing IRES puro
pWPI BLR	Trotard et al., 2016 [17]	Lentiviral transfer plasmid containing IRES BLR
3xnls-mScarlet-I	Addgene #98816	mScarlet red fluorescent protein nucleus
mScarlet-I-LaminB	Addgene #98831	mScarlet red fluorescent protein nuclear membrane
pmScarlet-i_Giantin_C1	Addgene #85050	mScarlet red fluorescent protein golgi apparatus
Lck-mScarlet-I	Addgene #98821	mScarlet red fluorescent protein plasmamembrane
pmScarlet-i_H2A_C1	Addgene #85053	mScarlet red fluorescent protein histone
pLifeAct_mScarlet-i_N1	Addgene #85056	mScarlet red fluorescent protein actin
pCMV3-2019-nCoV-Spike(S1+S2)-lo	SinoBiological; Cat#: VG40589-UT	SARS-CoV-2 (2019-nCoV) Spike wildtype
pcDNA3.3-SARS2-B.1.617.2	Addgene #172320	SARS-CoV-2 (2019-nCoV) Spike B.1.617.2
pcDNA3.3_SARS2_omicron_BA.1	Addgene #180375	SARS-CoV-2 (2019-nCoV) Spike BA.1
hCoV-19/Germany/BavPat1/2020-nucleocapsid	European Virology Archives: 026V-03883, EPI_ISL_406862	nucleocapsid source
Generated Plasmids		
pwpi 3xNLS-mScarlet	This paper	Lentiviral vector plasmids containing an mScarlet fluorescent protein tag
pwpi mScarlet-Lamin	This paper	
pwpi mScarlet-Giantin	This paper	
pwpi LCK-mScarlet	This paper	
pwpi H2A-mScarlet	This paper	
pwpi wt spike	This paper	Lentiviral vector plasmids containing a SARS-CoV-2 spike protein variant
pwpi delta-spike	This paper	
pwpi omicron-spike BA.1	This paper	
pwpi nucleocapsid	This paper	Lentiviral vector plasmid containing a SARS-CoV-2 nucleocapsid protein

Table S2. List of cell lines used or generated in the study

Name	Reference	Designation/Usage	Resistance
HeLa Kyoto		Human adenocarcinoma cell line	-
Embryonic kidney 293 T cells (HEK293T)		Human cell line for production of viral particles	-
HeLa mScarlet-3xNLS	This paper	Polyclonal cell lines constantly expressing mScarlet tagged to a unique subcellular localization	Blasticidin
HeLa mScarlet-Lamin	This paper		
HeLa mScarlet-Giantin	This paper		
HeLa mScarlet-LCK	This paper		
HeLa mScarlet-Lifeact	This paper		
HeLa mScarlet-H2A	This paper		
HeLa SARS-CoV2-spike Wuhan-Hu-1 strain	This paper	Polyclonal cell lines constantly expressing SARS-CoV-2 spike protein of a variant of interest	Puromycin
HeLa SARS-CoV2-spike delta	This paper		
HeLa SARS-CoV2-spike omicron BA.1	This paper		
HeLa SARS-CoV2 nucleocapsid	This paper	Polyclonal cell line constantly expressing SARS-CoV-2 nucleocapsid protein	
HeLa mScarlet-Giantin-Spike wt	This paper	Polyclonal cell lines constantly expressing mScarlet tagged to a unique subcellular localization and SARS-CoV-2 spike protein of a variant	Blasticidin + Puromycin
HeLa mScarlet-LCK-Spike delta	This paper		
HeLa mScarlet-H2A Spike omicron BA.1	This paper		
HeLa mScarlet-3xNLS SARS-CoV2-Nucleocapsid	This paper	Polyclonal cell line constantly expressing mScarlet tagged to a unique subcellular localization and SARS-CoV-2 nucleocapsid protein	

Table S3. List of used mAbs from Wang et al.[19] and their binding specificities

		**EC50 (ng/ml)***			
**ID in this study**	**Antibody ID** *Wang et al. 2022, PMID: 36149398*	**RBD Wuhan-Hu-1**	**RBD Delta**	**BA.1 spike**	**Binding category**
mAb 1	B2010	>1000	>1000	>1000	No variant
mAb 2	B2012	>1000	>1000	>1000	
mAb 3	B2000	4	19	38	All
mAb 4	B003	3	7	8	
mAb 5	B1073	2	>1000	6	All but Delta
mAb 6	B2005	3	>1000	33	
mAb 7	B1064	2	18	>1000	All but BA.1
mAb 8	B1072	4	7	>1000	
mAb 9	B1058	2	>1000	>1000	Wuhan-Hu-1 only
mAb 10	B2116	2	>1000	>1000	
mAb 11	B1005	4	353	>1000	Wuhan-Hu-1>Delta>BA.1
mAb 12	B026	15	451	>1000	
mAb 13	B2108	2	>1000	318	Wuhan-Hu-1>>Delta<BA.1
mAb 14	B1111	6	>1000	158	

*EC50 values taken from Wang et al. [19] Memory B cell responses to Omicron subvariants after SARS-CoV-2 mRNA breakthrough infection in humans. J Exp Med. 2022 Dec 5;219(12):e20221006. doi: 10.1084/jem.20221006. Epub 2022 Sep 23. PMID: 36149398; PMCID: PMC9513381.
